# Verification, Evaluation, and Validation: Which, How & Why, in Medical Augmented Reality System Design

**DOI:** 10.3390/jimaging9020020

**Published:** 2023-01-17

**Authors:** Roy Eagleson, Leo Joskowicz

**Affiliations:** 1 AI and Software Engineering Program, The University of Western Ontario, London, ON N6A 5B9, Canada; 2 School of Computer Science and Engineering, Edmond J. Safra Campus, The Hebrew University of Jerusalem, Givat Ram, Jerusalem 9190401, Israel

**Keywords:** system analysis, medical augmented reality, verification, evaluation, validation, uncertainty, observer variability, measurements accuracy

## Abstract

This paper presents a discussion about the fundamental principles of Analysis of Augmented and Virtual Reality (AR/VR) Systems for Medical Imaging and Computer-Assisted Interventions. The three key concepts of Analysis (Verification, Evaluation, and Validation) are introduced, illustrated with examples of systems using AR/VR, and defined. The concepts of system specifications, measurement accuracy, uncertainty, and observer variability are defined and related to the analysis principles. The concepts are illustrated with examples of AR/VR working systems.

## 1. Introduction

**Figure 1:**
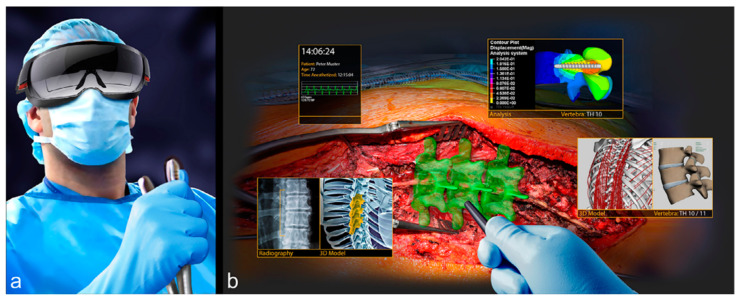
Augmented reality for spine surgery: (**a**) Surgeon with AR headset; (**b**) Augmented view showing various synthetic and augmented images.

This paper is a discussion about the fundamental principles of “Analysis” of Augmented and Virtual Reality Systems for Medical Imaging and Computer-Assisted Interventions (Figure 1). We contrast this with the “Synthesis” of AR/VR Systems and we raise this Analytic/Synthetic distinction foremost, because our aim is to emphasize the complementary role that both play, as they are both necessary for system design.

**Figure 2:**
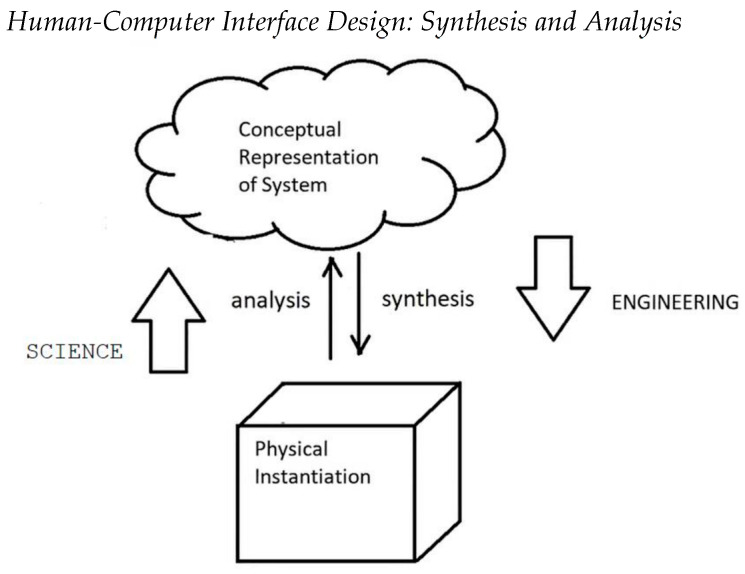
The analytic/synthetic distinction in the process of design.

To ground this in engineering design terms, “Synthesis” is the process whereby we proceed from system requirements to an implemented system which is comprised of hardware and software. Most engineering students are likely to be familiar with these concepts. However, within the same engineering design context, we emphasize that “Analysis” is the process whereby we take an implemented system and, through observation while the system is being utilized for the task for which it was designed, we compare its observed behavior and structure to those that were initially specified or extended through the process of design. This process is schematically shown in Figure 2.

We recognize that through the design process, it is natural and expected that the “requirements” themselves may change and grow. This is because the specification of requirements are often stated in ways that can be incorrect, inconsistent, ambiguous, or incomplete. Nevertheless, without some representation of system requirements, there would be no basis on which to form a system “Analysis”.

## 2. Verification, Evaluation, and Validation

Against that introductory backdrop, we foreshadow the intent of our paper: to discuss the scientific aspect of engineering design, based on taking requirements as sets of hypotheses for the structure and behavior of the system, and testing the implemented system accordingly. What will emerge is a distinction between “Verification”, “Evaluation”, and “Validation”—and, through examples, we will discuss these terms by considering motivational factors such as “What” they are, “How” we proceed, and “Why”.

Briefly stated, “Verification” is the process whereby any particular system requirement is observed to see whether or not it fulfils a specification (a binary measure). “Evaluation” measures how well the system can be used to fulfil a task—it is a performance measure, posed in terms of the speed and accuracy (or task time and error rate) for a suitably well-posed task. “Validation” calls into play considerations of internal vs external validity of the experiments, the sources of cognitive bias that may corrupt these measures, and questions regarding the elusive abstract entities (i.e., the “Constructs”) which we wish to measure but cannot be measured directly (e.g., “Surgical Skill”, “Anatomical Knowledge”, “Respect for Tissue”, etc.).

### 2.1. A Medical Augmented Reality System Design Examples

It will be instructive to start with a concrete example: we describe a recent project involving the design of an AR/VR system for computer-assisted spine surgery [1] (Figure 1). The authors define the context of their project as:

*“Augmented Reality (AR) is a rising technology gaining increasing utility in medicine. By superimposing the surgical site and the operator’s visual field with computer-generated information, it has the potential to enhance the cognitive skills of surgeons. This is the report of the first in man case with “direct holographic navigation” as part of a randomized controlled trial.”*

Their system specification can be stated precisely and informally: “A pointing instrument was equipped with a sterile fiducial marker, which was used to obtain a digital representation of the intraoperative bony anatomy of the lumbar spine. Subsequently, a previously validated registration method was applied to superimpose the surgery plan with the intraoperative anatomy. The registration result is shown in situ as a 3D AR hologram of the preoperative 3D vertebra model, with the planned screw trajectory and entry point for validation and approval by the surgeon. After achieving alignment with the surgery plan, a borehole is drilled and the pedicle screw is placed. Postoperative computer tomography was used to measure accuracy of this novel method for surgical navigation”.

The verification and evaluation of this system is stated in the overview as: “Correct screw positions entirely within bone were documented with a postoperative CT, with an accuracy similar to current standard of care methods for surgical navigation. The patient was mobilized uneventfully on the first postoperative day with little pain medication and dismissed on the fourth postoperative day”.

Finally, a statement that points to the continued investigations needed in order to attempt to “Validate” their measures in the context of a whole range of OR use cases and contexts: “This ‘first in man’ report of direct AR navigation demonstrates feasibility in vivo. The continuation of this randomized controlled study will evaluate the value of this novel technology”. Note that this overview paper was built upon the success of the engineering design efforts that were published in related papers [2,3,4,5,6].

### 2.2. An Example of the Validation of a Measurement System

One guiding principle that helps to properly define the terms is to emphasize that “Validity” is an attribute of an inference. When we make measures, they are used to draw conclusions; the validity of the inferences which we draw from those measures (which in turn can lead to conclusions) is what we are striving for. An inference is based on the evidence gathered from observations; one example of an observation is a quantitative measure. We could search the literature for a small set of papers which provide examples of validation of measurement systems; within the domain of biomedical engineering, we think that these are proper uses of the terms. These papers would share a characteristic in which each of them describes systems that are used to make physical measurements. We argue that this calls into play the notion of a ‘gold standard’—which is in a sense, a ‘specification’ on which verification, evaluation, and validation all become synonymous terms—but only for systems that are designed to perform physical measurements.

A recent paper [7] describes a camera-based system to measure the motion of the hand in a virtual environment; their system validity was “evaluated” based on “repeatability”, “precision”, and “reproducibility”. Their system “reliability” was estimated using a correlation with manual methods, a separate intraclass correlation coefficient on the measures, and a paired t-test and bias test. Note that the authors used these methods to gather evidence on the consistency of repeated measures, and to test for any difference between the depth camera and manual measurement procedures. The first word in their paper’s title is “Validity”. To be sure, their comparisons against alternate measurement systems can appropriately be called “concurrent validity”. When their paper suggests that the evidence was gathered “in the lab”, this might suggest that “out of the lab” measurements might differ. This raises the question of “internal validity” versus “external validity”. While this is described as a system “Validation”, there is no mention of ‘construct validity’, ‘content validity’, ‘external validity’, or even ‘face validity’. We suggest that this is because these extended and abstract forms of validity must be reserved for systems that are more complex than simple measurement systems.

We suspect that this is where much confusion enters the wider use of these terms. For systems that are designed to make physical measurements, ‘verification’, ‘evaluation’, and ‘validation’ all collapse more or less into synonymous terms. Let us now examine other domains in which systems are being designed as tools for accomplishing more complex tasks.

We have to take care, however; since while most would agree that ‘verification’, ‘evaluation’, and ‘validation’ are three separate levels of empirical investigation that need to take place as part of the whole design process for complex systems, there is some confusion in the literature as to what terms should be used to refer to them. One does not need to search far for examples of the terms being used in interchangeable ways. Therefore, while we move towards considerations of the use of “Validity,” let us explore this in conjunction with related terms “,Reproducibility, Replicability, Repeatability, and Reliability”.

### 2.3. Reproducibility, Replicability, Repeatability, and Reliability

In the context of the requirements and analysis of engineering systems, and in particular for medical devices, the concepts related to “Measurements” are “Accuracy”, “Precision”/“Recall”, and “Repeatability”, and “Reliability”. For “Specifications” derived from empirical values set by clinicians, the relevant concepts are to compare against a specified “Ground Truth”, and to consider inter- and intra- “Observer Variability”, under the more general term of “Uncertainty”. These terms are often confused, used as synonyms, and even misunderstood. We will briefly define the terms here and later, and illustrate them with examples. No matter how you slice or dice the terms, the following three seem to come up as synonyms: reproduce, replicate, and repeat.

The terms stack up (i.e., our “R”-terms will stack up in a hierarchy, just as our “V”-terms). Consider that if measurements are not repeatable, then they cannot be reliable. If measurements are not reliable, then experiments cannot be replicated, i.e., they are irreproducible.

For example, for the AR/VR system for computer-assisted spine surgery described above (Figure 1), the main specification is the desired accuracy of the final screw position with respect to the preoperative plan (ground truth). Some of the main relevant measurements are: position of the screws in the preoperative plan, registration accuracy of the planned screw positions with respect to the acquired pedicle digital representation (previously validated), accuracy and precision of the tracked instrument and of the drilled borehole alignment with the preoperative plan, and postoperative. The observer variability is the difference of the desired preoperative screw position as defined by two or more clinicians, e.g., the pedicle screw angle and screw depth. The uncertainty is the target and range of values that are set as specifications by the system designer and the clinicians, e.g., the pedicle screw angle within one degree of the target value. Both the observer variability and the uncertainty can be both patient-specific (a single patient) or the mean of a set of patients (patient cohort).

**Figure 3:**
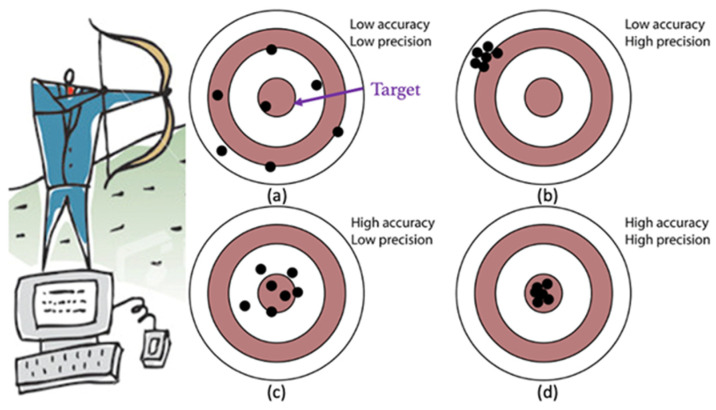
Classical illustration of accuracy and precision: an archer (left) repeatedly shoots at a predefined target (ground truth). Each black denotes an independent measurement. Four scenarios are possible: (**a**) low accuracy and low precision; (**b**) low accuracy and high precision; (**c**) high accuracy and low precision; (**d**) high accuracy and high precision.

The terms accuracy and precision are well known and have precise definitions, cf. Wikipedia (Figure 3). Accuracy quantifies the difference between observed or measured values and what was established as the true value, also called ground truth. Precision is how close the measurements are to each other. Accuracy measures systematic errors, i.e., the statistical bias of a given measure from a mean value. Precision measures the statistical variability of random errors. A measurement can be accurate or not, precise or not, or neither.

**Figure 4:**
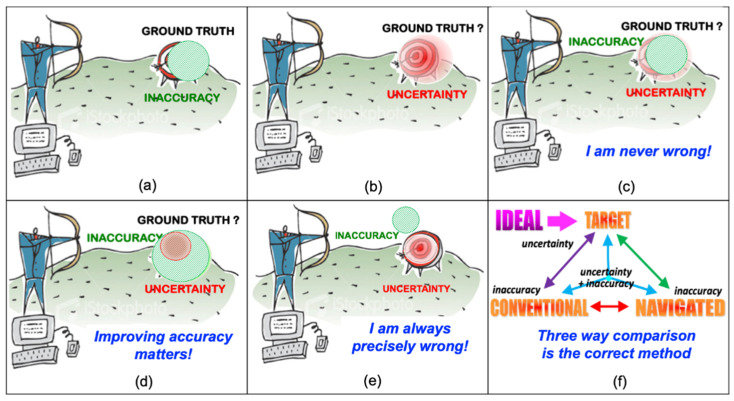
Illustration of the relation between ground truth, uncertainty, and accuracy: (**a**) the target ground truth is the specification with its mean value (center of the target) and deviations from it (concentric circles); the location of the shots (green circle) define the accuracy; (**b**) the target ground truth is uncertain (red circle); (**c**) when the uncertainty of the target ground truth is larger than the accuracy of the shots (green circle), the specifications are always met (“I am never wrong”); (**d**) when the uncertainty of the target ground truth is smaller than the accuracy of the shots, improving the system accuracy specifications are not always met (“Improving accuracy matters”); (**e**) when the accuracy of the shots improves but have a bias, the system accuracy specifications will never be met; (**f**) evaluation scheme of a procedure performed by conventional means and a computer assisted one.

We now discuss the specifications and their relation to the ground truth, to uncertainty, and to accuracy. For this purpose, we will use the analogy of an archer shooting at a target (Figure 3 and Figure 4). When the target ground truth is and defined as the specification with its exact mean value (center of the target) and deviations from it (concentric circles), the accuracy and precision are as defined before (Figure 4a). However, when the mean ground truth value is not precisely known, as it is often the case for medical specifications as a result of uncertainty or observer variability in defining them, the ground truth target location is shifted (Figure 4b). In this case, the uncertainty affects the accuracy. However, when the uncertainty of the target ground truth is larger than the accuracy, the specifications are always met (Figure 4c). In this case, improving the accuracy of the system or defining tighter specifications is unnecessary, as it will not affect the outcome. However, when the uncertainty of the target ground truth is smaller than the accuracy, the specifications are not always met (Figure 4d). In this case, improving the accuracy of the system or defining tighter specifications does make a difference and should be carefully considered. However, when the accuracy is improved but has a bias, the specifications will never be met (Figure 4e).

These scenarios have important consequences when performing the analysis of a system and its comparison with a conventional procedure (Figure 4f). For simplicity, assume that we focus on a single real-valued parameter, e.g., the pedicle screw angle in the AR/VR system for spine surgery shown above (Figure 2). For a given patient and procedure, this angle has an ideal value, that is, the angle for which the pedicle screw is most beneficial/best performs its job. In most cases, this angle cannot be known precisely, as it depends on many complex and interrelated phenomena. Instead, we choose a target value. This is the empirical value that, in the opinion of the treating physicians, will yield the best results for the patient. The target value, in our case the target pedicle screw angle, is in fact a range—the interval defined by the minimum and maximum acceptable value. This interval reflects the uncertainty that is associated with the target value.

Now, consider two possible ways to achieve the target value: the conventional procedure, e.g., in our example the manual orientation of the pedicle screw hole drill (conventional), and the augmented reality navigated method (navigated). To establish the accuracy of each method independently, the achieved values of repeated trials with the method are compared to the target value—the mean value, measured value, standard deviation, and minimum and maximum values. When the measured values interval is fully included in the target value uncertainty interval, the method is deemed accurate. The intervals overlap defines the measure of accuracy.

Consider now the comparison between the conventional and the navigated procedure. One approach would be to directly compare the mean values and intervals for each method. However, this two-way comparison does not take into account the target value and its uncertainty interval. Thus, even when the interval of the navigated procedure is inside the interval of the conventional procedure, which may indicate that the navigated procedure is more accurate than the conventional procedure, it can still be away from the target value and/or have little overlap with its uncertainty interval.

**Figure 5:**
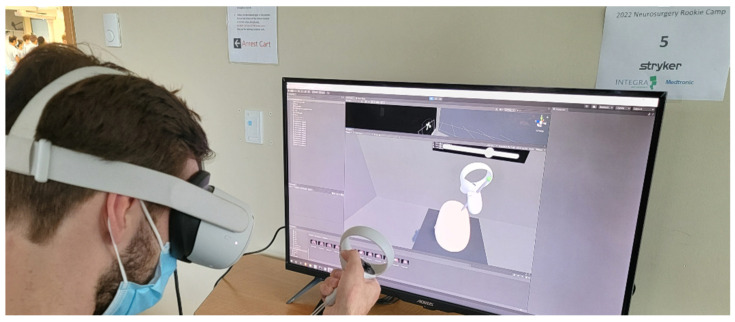
Illustration of a VR-based neurosurgery simulator.

**Figure 6:**
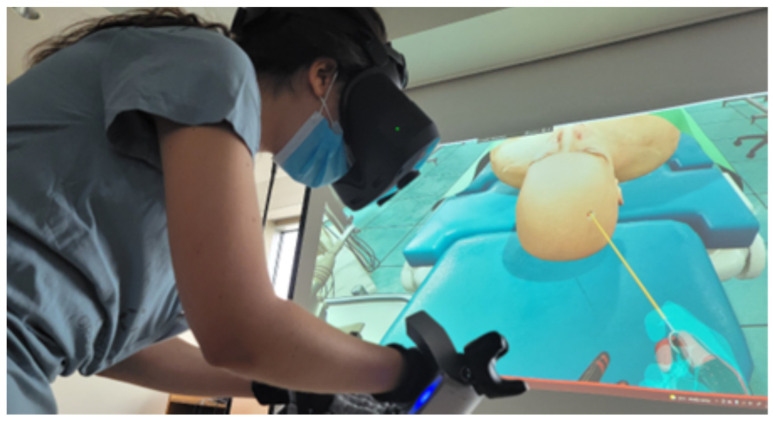
Illustration of an AR-based neurosurgery simulator.

This illustration leads us to postulate that the correct way to compare the performance accuracy of two procedures—manual and navigated in our examples (cf. Figure 5 and Figure 6)—is three-way comparison of the target value and its uncertainty, the conventional and the navigated, and procedure accuracies and measurement inaccuracies. Measures can be biased and/or uncertain. Distinguishing the two kinds of error is important, but we would also need their task time, in order to fully Evaluate their Performance. Note that ‘Performance’ is the product of speed and accuracy.

We now relate the concepts of uncertainty and accuracy to the three elements of the analysis: verification, evaluation, validation.

***Verification:*** The ideal and target values, and their associated uncertainty, are directly relevant for the verification since they are the values to which the experimental results values will be compared to determine the pass/fail criteria. The verification must take into account the uncertainty of the target value. Note that the verification does not involve comparison with other ways of performing the procedure, e.g., conventional. Thus, when validating the AR system described above (Figure 2), there is no need to collect and analyze results from the conventional procedure.

***Evaluation:*** since it measures how well a system can be used to fulfil a task, evaluation necessarily involves the three-way comparison with the conventional method. It must take into account the both the target uncertainty and the accuracy of the conventional and the navigated procedure. Thus, when *Evaluating* the AR/VR System for spine surgery described above (Figure 2), there is a need to collect and analyze results from both the conventional and the navigated procedures.

***Validation:*** since validation calls into play considerations of internal vs external validity of the experiments, the sources of cognitive bias that may corrupt these measures, and other user-related issues, e.g., surgical skills and anatomical knowledge, mostly involves the new system and less the conventional one. Thus, a two-way comparison with the target and with additional considerations is in order. The uncertainty and accuracy are relevant and part of the validation, and come together with other criteria. It is only when these criteria are stated with respect to the conventional procedure that the three-way comparison is in order. Thus, for example if an improvement of surgical skills or a reduction in operating time is stated, then these measures must be determined both for the navigated and for the conventional procedures.

## 3. Recommendations

In a recent MICCAI workshop on Ethical and Philosophical Issues in Medical Imaging (EPIMI’2022) [8], the opening keynote introduced the need for these same three phases. What kind of evidence do we need to gather in order to justify the use [of systems]? This also raises questions around whether measures can be biased or ‘fair’, and are they generally well-understood or explainable? Finally, the fundamental question of whether the measures lead to good decisions or valid inferences (it was on this last basis that Cronbach and colleagues [9,10,11] established a framework for ‘validity’). On the basis of McCradden’s talk, which we feel were informed by Cronbach, et al., we can reconstruct the following items, in the context of AR/VR systems design:

− What are appropriate measures that need to be gathered as evidence?
− Are the measures fair or biased? (uncertainty, accuracy, and precision?)
− Are the measures well-understood and explain outcomes?
− Can the measures be used to form *valid inferences* about the outcomes?

We all face concerns about how evidence may be gathered within experimental settings, yet they might not generalize or extrapolate to real-world situations. From our perspective, this exposes the distinction between the internal validity vs the external validity of evidence gathering. Indeed, we contend that this reveals a fundamental trade-off in experimental design: the more one attempts to control the experimental parameters, the higher the internal validity but the lower the external validity.

In contrast, when one attempts to make the experiment as “real world” as possible, there is an explosion in the number of free parameters that need to be systematically controlled; this leads to an infeasible experimental operationalization and analysis. McCradden (2022) [12] suggests that the ‘real’ environment may be different from the controlled ‘in silico’ environment in which it was tested. However, instead of saying ‘tested’ she says ‘validated’ in this context.

Our contention is that ‘validation’ is a reserved word indicating that the external context, including patient outcomes and even concerns of ‘fairness’, are the broader domain with considerations of ‘validity’.

We contend that evidence-gathering from ‘in silico’ experiments can allow us to “Verify” system design, and also to “Evaluate” the performance of the system as a tool to perform a specified task. In other words, we need technical “Verification” and task-based performance “Evaluation”.

Considerations for whether the experimental task has any relationship to the real-world task is a question about “Content Validity” and begins to allow some convergence towards the more difficult questions surrounding “Construct Validity”.

We concur with the approach taken on this project, and use this as a basis for pointing out how some of these terms can be misunderstood—or at least used with crossing terms—in our literature. Furthermore, as we are considering making observations of a system, some attention must be cast upon the potential sources of error on these measures, as well as the particular meanings of these observations when aggregated.

Any evidence gathered using AR/VR systems—whether that be for diagnosis, or to be used for guiding clinical procedures—may be done so in a way which is non-specific to the cases for which it was designed. Indeed, that information may be hidden from the end users of the technology.

Consider this as foreshadowing a potential problem that we might associate with “Training Download”. If we get to a stage where tools are disseminated, perhaps using online technologies such as MOOCs or cloud-based deployment to remote headsets, we risk having an inappropriate mapping between the kind of user for which the system was designed, and the actual population of users employing the technology.

This kind of mis-matching cannot be picked up at the system verification stage, nor is it likely to be identified as a problem at the system performance evaluation (which will involve only a limited subset of the user population).

It becomes a concern where the validity of inferences drawn from the measures that are logged and analyzed. Remember: it is not a system which is validated, it is the inferences drawn from measures gathered through usage of the system which can be examined, and considerations of validity can be discussed.

## 4. Discussion

We have attempted to expose the distinction between these levels of empirical work. We also emphasized that all three must be done as part of the design of systems and, in particular, of augmented and virtual-reality systems; whether they be used for diagnostics, planning, or training. We emphasize that there must be three levels of evidence-gathering, on the empirical side of the overall design process.

One level is where the designers check the system to verify whether the system (and its sub-components) meet their specifications.

The second level is where experiments are carefully set up to observe the performance of users—on a range between experts and novices—in order to quantify their performance in terms of the speed and accuracy with which they can accomplish certain tasks. Taken together, they form a curriculum that can arguably span the content that would be needed in order to gain clinical skills that can improve patient outcomes.

The third level is to consider all of the evidence gathered, and to compare and contextualize against the backdrop of responsible and like-minded research. Only by considering the validity of the inferences drawn from experimental measures, can a discussion begin about whether any particular system design is worthwhile.

Consider that we agree with the positions that McCradden et al. (2022) [12] takes in her arguments. Rather than focus on her particular word-sense of these terms, we agree that there should be evidence gathering at the basic technical level, at the performance level when the system is being used to perform an intended task, and at the more general and fundamental level in the context of the broader sense of patient outcomes.

Even within the popular press, there is a rising sentiment that there should be more empirical work done, in consideration of the systems to be used for any decision-making that would involve systems for “Medical Imaging, and to be sure, for computer-assisted interventions. The front level is system-centric, the second is about task-based performance, and yet, there is a third level which is much more intangible, yet more important. Therefore, it deserves the additional effort and clinical trials in order to pose questions of whether a system is worthwhile.

We are not so pedantic to suggest that we can stop any particular group from using whichever term they want. You can call them phase-1, phase-2, and phase-3 if you want. Who makes the rules? It is not clear. “Artificial Intelligence” should never have been co-opted as a term to refer to “Inferences drawn using systems trained using Machine Learning”. If you want to call a “virtual environment” a “hologram”, go ahead! Even though the virtual environment that you see with a HoloLens is *not* a “hologram”, that is a reserved word, and that term should never have been co-opted through casual hyped usage. The same goes for “Validation”, “Evaluation”, and “Verification”.

We simply close by recommending proper and principled usage of the terms “Verification”, “Evaluation”, and “Validation”, and we have argued principally for why these levels must exist, and why they ought to be referred to as such! In any case, the end-goal is the same: to improve healthcare outcomes by emphasizing the importance of the empirical (analytic) side of the design process, which will require just as much energy (if not more) than the synthesis side... no matter what language we speak.

## Data Availability

Not applicable.
